# MinION Nanopore‐Enabled Identification and Genomic Characterization of *Pseudomonas syringae* Complex Infecting Blueberry Using Symptomatic Plant Samples and Pathogen Pure Culture

**DOI:** 10.1002/cpz1.70329

**Published:** 2026-02-25

**Authors:** Rishi R. Burlakoti, Sanjib Sapkota, Pragyan Burlakoti, Mark Lubberts, Amy Novinscak, Harvinder Bennypaul

**Affiliations:** ^1^ Science and Technology Branch, Agassiz Research and Development Centre Agriculture and Agri‐Food Canada Agassiz British Columbia Canada; ^2^ British Columbia Ministry of Agriculture and Food Abbotsford British Columbia Canada; ^3^ Science and Technology Branch, Summerland Research and Development Centre Agriculture and Agri‐Food Canada Summerland British Columbia Canada; ^4^ Centre for Plant Health Sidney Laboratory Canadian Food Inspection Agency North Saanich British Columbia Canada

**Keywords:** blueberry, disease diagnostics, nanopore, *Pseudomonas syringae* complex, whole‐genome sequencing

## Abstract

*Pseudomonas syringae* complex (Psc) causes bacterial blight and canker on blueberry and other fruit crops. Reliable and cost‐effective pathogen detection is essential for managing crop diseases. The Oxford Nanopore MinION has emerged as a powerful tool in plant disease diagnostics and genetic characterizations of plant pathogens due to streamlined library preparation protocols, rapid sequencing turnaround, the ability to generate long reads, and enhanced taxonomic resolution. However, standardized and reproducible methods for whole‐genome sequencing of the plant pathogenic Psc remains limited, especially for pathogens infecting blueberry tissues. Therefore, we describe several protocols including DNA extraction and quality control from both pure bacterial cultures and infected blueberry tissues (e.g., infected leaves and stem tissues), library preparation, and whole‐genome sequencing using the MinION platform. The challenges of working with low bacterial pathogen biomass in plant tissue, contamination from host DNA, and sample variability are addressed through optimized workflows and troubleshooting strategies. Furthermore, we provide a modular bioinformatics software pipeline ranging from user‐friendly EPI2ME tools to command‐line analyses with Kraken2 and Krona for genome assembly and annotation and taxonomic identification, and comparative and evolutionary studies of Psc. These methods provide a reproducible framework for the genomic characterization and diagnostics of the Psc in the context of agricultural disease diagnostics, with the potential flexibility for adaptation to other bacterial taxa and sample types. © 2026 His Majesty the King in Right of Canada. *Current Protocols* published by Wiley Periodicals LLC. Reproduced with the permission of the Minister of Agriculture and Agri‐Food.

**Basic Protocol 1**: Isolation of pseudomonad cultures from infected plant tissues

**Basic Protocol 2**: DNA extraction from pure cultures of pseudomonads

**Basic Protocol 3**: DNA isolation from infected host tissues

**Basic Protocol 4**: Quality control, library preparation, and whole‐genome sequencing

**Basic Protocol 5**: Taxonomic identification using EPI2ME Desktop application

**Support Protocol**: Taxonomic identification using Kraken2 and Krona in Linux

**Basic Protocol 6**: Bioinformatics analyses for genome assembly and annotation

## INTRODUCTION

Accurate and timely diagnosis of crop diseases, along with the characterization of plant pathogenic bacteria, is vital for safeguarding agricultural productivity, biosecurity, and international trade. *Pseudomonas syringae* complex (Psc) and other pseudomonads are major plant pathogenic bacteria infecting several high‐value crops, including fruits, vegetables, and ornamental species. Strains within Psc was previously referred as *P*. *syringae*, but Gomila et al. (2017) reclassified and categorized several phylogenomic species within Psc. Three phylogenomic species (*Pseudomonas avellanae, P. syringae*, *Pseudomonas viridiflava*) of Psc were identified to cause blight and canker in highbush blueberry in British Columbia, Canada (Latchman et al., 2026). Plant pathogenic bacteria including pseudomonads were traditionally identified and characterized using pathogen morphology, biochemical characterization, and PCR‐based or Sanger sequencing. These traditional diagnostic approaches are valuable but have limitations in their resolution and reproducibility. For example, pathogen identification using Sanger sequencing targeting conserved regions of the 16S rRNA gene often lacks the taxonomic resolution necessary to distinguish closely related species of *Pseudomonas* as well as phylogenomic species of Psc. Available PCR‐based assays (Guilbaud et al., 2016) also cannot identify the phylogenomic species of the Psc. These methods may not be applicable to amplify the pathogen DNA directly from plant tissue. Morphological or biochemical methods may not be sufficient to identify the bacteria at species level (Gomila et al., 2017).

Recent advances in high‐throughput sequencing (HTS) technologies have transformed the field of pathogen diagnostics by enabling unbiased, high‐resolution analysis of microbial genomes. Among these, Oxford Nanopore Technologies (ONT) offer unique advantages for pathogen detection and genomic characterization. The MinION platform enables real‐time data acquisition, long‐read generation, and rapid library preparation in a compact and portable format, making it suitable for both field and laboratory diagnostics. These features allow for the simultaneous detection of multiple pathogens, including unculturable or previously uncharacterized taxa, directly from infected plant tissues, without the need for successful pathogen isolation (Haveman et al., 2022; Shivashakarappa et al., 2022). Despite these advantages, comprehensive and standardized ONT‐based protocols for isolation of plant pathogenic bacteria, extracting high quality DNA from host‐associated tissues, and whole‐genome library preparation for plant pathogenic pseudomonads remain limited.

In this article, we present a series of optimized laboratory and computational steps, developed in our laboratory, to study pseudomonads infecting blueberry using whole‐genome sequencing via Oxford Nanopore's MinION platform. These methods have been refined for genomic sequencing for both pure bacterial cultures as well as directly from infected host tissues. The protocols include detailed procedures for isolation pseudomonad cultures (Basic Protocol 1), DNA extraction from pure cultures of pseudomonads (Basic Protocol 2) and infected plant tissues (Basic Protocol 3), and quality control and library preparation for ONT sequencing (Basic Protocol 4). While our protocols focus on Oxford Nanopore sequencing, the methods (Basic Protocols 1 to 3) are also adaptable to other platforms, including Illumina sequencing.

As in microbiome research, reproducibility and interpretability remain key challenges in HTS‐based diagnostics. Our suite of protocols addresses these concerns by incorporating positive controls (e.g., known pathogens to verify extraction, library preparation, and sequencing) and negative controls (e.g., blank extractions and non‐template libraries to detect potential contamination). To support users with varying levels of computational expertise, we provide two tiers of a bioinformatic pipeline: a user‐friendly, GUI‐based approach using EPI2ME (Basic Protocol 5), and a more customizable command‐line pipeline integrating Kraken2 and Krona (Support Protocol). Both pipelines enable rapid and accurate taxonomic identification, while Krona plots offer high‐resolution visualization of complex or mixed infections. In addition, we present a protocol for genome assembly and annotation of pure bacterial isolates (Basic Protocol 6), providing a tool for comparative and evolutionary studies of plant‐pathogenic pseudomonads. These tools will empower researchers to convert raw sequencing data into actionable insights for pathogen surveillance and disease management.

For reproducibility and confidence in the results obtained by the proposed protocols, we emphasize key considerations for sample collection and tissue selection, the inclusion of appropriate controls, the use of custom reference databases to capture diverse microbiomes, and best practices for interpretation and reporting of taxonomic classification results. By integrating optimized wet‐lab protocols with accessible bioinformatics workflows, we provide a scalable and portable sequencing solution. This approach enables real‐time detection of pseudomonads including Psc directly from blueberry samples from fields. In addition, the protocols developed in this study can be broadly transferable to other bacterial taxa and crops with some modifications.

## Basic Protocol 1 ISOLATION OF PSEUDOMONAD CULTURES FROM INFECTED PLANT TISSUES

Here we describe optimized methods for isolating plant‐pathogenic pseudomonads from symptomatic plant tissues, including blueberry leaves, stems, and floral buds. This protocol was used in our recent studies (Latchman et al., 2026). For maximizing chances of detecting an infection, it is essential to collect samples from all over the affected plant and, preferably, from multiple regions of a field to capture potential spatial variation in pathogen distribution.

If immediate processing is not feasible, enclose plant samples in air‐tight bags (e.g., Ziploc plastic bag) and store at 4°C to preserve pathogen viability and avoid saprophytic growth of other microbes. If moisture is present in plant samples, it can be absorbed and removed using paper towel before storing at 4°C. Prior to processing, perform a visual inspection of the sample—either with the naked eye, a magnifying lens, or a stereo microscope—and record relevant symptoms, such as necrosis, discoloration, tissue distortion, stunting, wilting, etc.

*NOTE*: All isolation procedures must be conducted inside a certified biological safety cabinet (BSC) to maintain sample integrity, ensure user safety, and prevent environmental contamination. This controlled environment is essential for maintaining the reliability of downstream analyses, including DNA extraction and whole‐genome sequencing.

### Materials

Infected or symptomatic plant tissue
10% bleach
Tween 20 (Thermo Fisher, cat. no. 003005)
H_2_O, sterile distilled
70% ethanol
King's B medium (see recipe)

Personal protective equipment (PPE), including laboratory coat, nitrile gloves, protective eyewear
Sieve and clip
BSC (NuAire)
Sterile Petri dishes (VWR, cat. no. CA25389‐328)
Forceps (VWR, cat. no. 82027‐386)
Scalpels (VWR, cat. no. 76457‐516)
Sterile inoculating loops (Thermo Fisher Scientific, cat. no. R50193)
Bunsen burner (VWR, cat. no. 470148‐926)
Parafilm or sealable plastic bags (VWR, cat. no. 102091‐164)

#### Tissue selection and surface sterilization

1 Before starting, wear appropriate PPE including a lab coat, gloves, and safety goggles.
2 Select symptomatic tissue from distinct lesion areas, including the advancing edge of lesions and a small portion of healthy tissue.
3 Using a sieve, gently wash the selected leaf or stem tissue samples under running tap water to remove surface debris.
4 Prepare all required materials in a BSC to maintain sterile conditions and workflow efficiency.
  a. Place one sterile Petri dish containing ∼30 ml of 10% commercial bleach with a drop of Tween 20 for surface sterilization of plant tissue, along with three additional Petri dishes filled with sterile distilled water for sequential rinsing of plant samples.
  b. Set out one labeled sterile Petri dish containing ∼1.5 ml of sterile distilled water for crushing plant tissues.
  c. Include a container with 70% ethanol for disinfecting tools, and ensure that sterile forceps, a scalpel, and an inoculating loop are within reach and placed aseptically inside the BSC.
5 Transfer the washed tissue into the 10% bleach + Tween 20 plate inside the BSC for up to 30 s for surface sterilization.
6 Rinse the sterilized tissue sequentially in three separate sterile distilled water Petri dishes, allowing 2 min in each rinse.
  After each rinse, flame‐sterilize the forceps by dipping them in 70% ethanol, briefly passing through the Bunsen burner flame, and allowing them to cool before reusing.
  Do not reinsert hot forceps into ethanol (fire and shattering hazard).

#### Tissue maceration

7 Place sterilized plant tissues (4 to 5 pieces) into the Petri dish containing 1.5 ml water and cut tissue samples into small pieces (<0.5‐mm sizes) with sterile forceps and scalpel. Ensure that cut plant tissues are dipped in water.
8 Cover plate and allow bacterial suspension to diffuse for 30 to 40 min. Record start time on plate lid.
9 Label two isolation medium plates as follows on the bottom of the plate: Sample no. – replicate no. – host – date and medium type (e.g., 1000‐1 – Blueberry – 2025‐Aug‐07 – KB).

#### Plating and streaking

**Figure 1:**
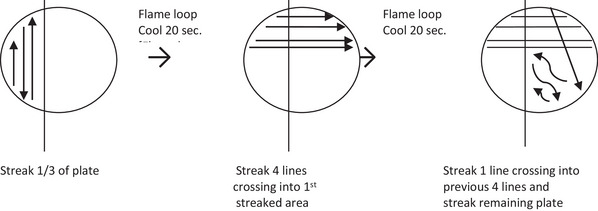
Streaking of macerated tissue suspension for bacterial culture isolation.

10 Sterilize loop (flame until red‐hot, cool 20 s by touching in medium).
11 Streak loopful of macerated tissue suspension across ∼⅓ of the King's B medium surface (Fig. 1).

12 Flame and cool loop again. Streak four lines across previous streak.
13 Flame and cool loop again. Streak a final line into last streak, covering the remaining plate.
14 Seal plates with Parafilm and place upside down in sealable plastic bag.

#### Incubation

15 Incubate plates at 22° ± 2°C by keeping them in an inverted position to avoid condensation.
16 Check daily for bacterial growth.

#### Subculturing

17 Select well‐isolated colonies for subculture. Label new plate: Sample no. – colony no. – host – date [e.g., 1000‐1–1 Blueberry – 2025‐Aug‐07 (colony 1 from plate 1)].
  Subculturing should be repeated until morphologically uniform colonies are obtained.
18 Flame‐sterilize loop, cool, and pick colony. Avoid contact with neighboring colonies.
19 Streak onto fresh medium as described above (steps 11 to 14).
20 Incubate at 22° ± 2°C and observe for pure culture growth.
21 Store original isolation plate in cold storage.

## Basic Protocol 2 DNA EXTRACTION FROM PURE CULTURES OF PSEUDOMONADS

This protocol outlines a rapid, reliable, and cost‐effective method for extracting genomic DNA from pure cultures of *Pseudomonas* spp. using the Bio‐Rad InstaGene Matrix. The procedure minimizes enzymatic degradation and yields PCR‐ready extracts suitable for downstream applications including sequencing.

### Materials

InstaGene Matrix (Bio‐Rad, cat. no. 7326030)
Pure culture of *Pseudomonas* spp. on solid medium (Plant Pathology laboratory at Agassiz Research and Development Centre, AAFC)
70% ethanol

PPE including laboratory coat, nitrile gloves, protective eyewear
BSC (NuAire)
Digital heat block or water bath (VWR)
Vortex mixer (VWR)
Sterile toothpicks
1.5‐ to 2.0‐ml microcentrifuge tubes (locking caps recommended)
Pipettes: 10 to 100 µl and 100 to 1000 µl
Filtered pipette tips
Filtered pipette tips with wide opening for InstaGene
ThermoMixer (Eppendorf)
Microcentrifuge (IEC Micro CL17, Thermo Fisher Scientific)
–20°C freezer (for long‐term DNA storage)
4°C refrigerator (for short‐term storage)
Timer

#### Sample preparation

1 Before starting, wear appropriate PPE. Perform all colony handling steps inside a BSC to maintain aseptic condition. Preheat the digital heat block to 100°C or hot water bath with range from 0° to 100°C. Vortex the InstaGene Matrix thoroughly before use to ensure even suspension.
  Include positive (e.g., known pathogens to verify extraction) and negative extraction controls (e.g., blank extractions tube) for quality assurance.
2 In the BSC, pick a single well‐isolated colony from a pure pseudomonads culture using a sterile toothpick.
3 Transfer the colony to the inside wall of a labeled microcentrifuge tube (1.5‐ to 2.0‐ml, locking cap).
  Repeat for all samples, including positive control. Spray gloves and disinfect work area with 70% ethanol between samples.

#### Cell lysis and DNA release

4 Using a pipette, add 200 µl InstaGene Matrix to each sample tube, including controls.
  InstaGene matrix should be mixed at moderate speed on a magnetic stirrer to maintain the matrix in suspension.
5 Place tubes in the ThermoMixer set at 56°C and 40 × *g* or a hot water bath or heat block at 56°C for 20 min.
6 After incubation, vortex tubes at high speed for 10 s.
7 Immediately transfer tubes to the 100°C heat block for 8 min.
  Place a weight on top of the closed tubes to prevent accidental opening of the caps at high temperature, causing the liquid to spill out.

#### DNA recovery and storage

8 Vortex tubes at high speed for 10 s, then centrifuge 2 min at 14,400 × *g*, room temperature.
9 Carefully transfer ∼150 µl supernatant (containing genomic DNA) into a new labeled microcentrifuge tube.
  Labels should include DNA, submission no., tissue type, and pathogen tested.
10 Store DNA extracts at –20°C for long‐term storage, or at 4°C for short‐term storage.

## Basic Protocol 3 DNA ISOLATION FROM INFECTED HOST TISSUES

This protocol details the extraction of genomic DNA from blueberry tissues infected with *Pseudomonas* spp. using the Qiagen DNeasy Plant Mini Kit with some modifications in sample preparation and lysis of the plant tissue. The procedure is designed to maximize yield and purity from plant tissues that often contain high levels of polysaccharides and secondary metabolites, which can inhibit downstream applications.

### Materials

DNeasy Plant Mini kit (Qiagen, cat. no. 69104)
96 to 100% ethanol
Infected plant tissue
100 mg/ml RNase A (Qiagen, cat. no. 19101)
Lysing Matrix A, 2‐ml tubes (MP Biomedicals, cat. no. 1169130‐CF**)**

PPE including laboratory coat, nitrile gloves, protective eyewear (if needed)
Digital dry block heater or water bath (VWR)
BSC (NuAire)
Forceps (VWR, cat. no. 82027‐386)
Scalpels (VWR, cat. no. 76457‐516)
Bunsen burner (VWR, cat. no. 470148‐926)
Pipettes: 0.5 to 10 µl, 10 to 100 µl, and 100 to 1000 µl
Filtered pipette tips
TissueRuptor (e.g., Omni Bead Ruptor, VWR)
ThermoMixer (Eppendorf)
Microcentrifuge (Thermo IEC Micro CL17, Thermo Fisher Scientific)
Biohazard waste bags (VWR, cat. no. 77778‐352)
1.5‐ microcentrifuge tubes
–20°C freezer (for long‐term DNA storage)
4°C refrigerator (for short‐term storage)
Vortex mixer (VWR)

#### Sample preparation

1 Prior to starting, wear appropriate PPE. If necessary, precipitates in buffers AP1 and AW1 from the DNeasy kit should be redissolved before use, and ethanol must be added to the AW1 and AW2 buffer concentrates as indicated by the manufacturer. Preheat a water bath or heating block to 65°C.
2 Sample preparation involves collecting ∼0.1 g of symptomatic tissues from distinct lesion areas, including the advancing edge of lesions and a small portion of healthy tissue. In a BSC, sterilize forceps and scalpel by dipping in 70% ethanol and flaming, then allowed to cool before transferring the tissue into Lysing Matrix A tubes.
  Gloves should be changed or sprayed with 70% ethanol in between samples to prevent cross‐contamination.
  Include appropriate extraction controls (positive and negative).

#### Lysis

3 Using pipettes add 400 µl buffer AP1 and 4 µl RNase A (100 mg/ml) to Lysing Matrix A tube.
  Do not mix Buffer AP1 and RNase before use.
4 Lyse tissue in TissueRuptor at Speed 6.0 for 40 s.
5 Following homogenization, incubate samples tube in the ThermoMixer set to 65°C and 40 × *g* for 10 min, or in the water bath or digital heat block set to the same temperature and time. If incubating samples in water bath or heat block, mix the samples regularly at every 2 to 3 min by inverting the tubes manually.

#### Precipitation and clearing

6 Add 130 µl of buffer P3 to the lysate, mixing by inversion and incubate at 4°C for 5 min to precipitate impurities.
7 Centrifuge sample tubes for 5 min at 17,500 × *g*, room temperature, to pellet debris.
8 Transfer supernatant to QIAshredder Spin Columns (lilac) placed in labeled 2‐ml collection tubes. Ensure the caps on the columns are closed tightly. Centrifuge for 2 min at 17,500 × *g*, room temperature.
9 Discard the lilac QIAshredder Spin Columns in a biohazard bag, saving the bottom 2‐ml collection tubes.
10 Pipette the entire flow‐through fraction (450 µl) from the 2‐ml collection tubes onto a new 1.5‐ml sterile tube, transferring without disturbing the debris pellet, if formed.
11 Add 1.5 volumes of buffer AW1 solution (e.g., 675 µl) directly to the cleared lysate and mix immediately by pipetting.

#### DNA binding

12 Transfer 650 µl mixture (including any precipitate) to DNeasy Mini Spin Column in 2‐ml collection tube. Centrifuge for 1 min at 10,000 × *g*, room temperature, and discard flow‐through.
13 Repeat with remaining lysate.

#### Wash

14 Place spin column in new 2‐ml collection tube. Add 500 µl buffer AW2 and centrifuge 1 min at 10,000 × *g*, room temperature.
15 Repeat wash with 500 µl AW2 in new collection tube.
16 Centrifuge 2 min at 17,500 × *g*, room temperature, to dry membrane.
17 Carefully transfer column to labeled 1.5‐ml microcentrifuge tube (including submission no., sample type, PCR test abbreviation, date for controls).
  For controls, tubes are labeled with NEC and/or PEC and the date instead of the submission no. and sample type. The date on the extraction controls ensures it has a unique identity.
  It is good practice to label the sides of each tube with an identifier in case the lid breaks off during the final spin.

#### DNA elution and storage

18 Add 100 µl buffer AE directly to column membrane.
19 Incubate 5 min at room temperature (15° to 25°C) followed by centrifugation for 1 min at 6400 × *g*, room temperature.
20 DNA extracts are stored either at –20°C for long‐term preservation or at 4°C if used for short‐term storage.

## Basic Protocol 4 QUALITY CONTROL, LIBRARY PREPARATION, AND WHOLE‐GENOME SEQUENCING

This protocol outlines the quality control steps and preparation of DNA libraries from purified *Pseudomonas* genomic DNA, derived both from pathogen pure cultures and directly from infected host plant tissues, for whole‐genome sequencing using the Oxford Nanopore MinION platform. Library preparation utilizes the Native Barcoding Kit 24 V14, enabling multiplexing of up to 24 samples per sequencing run. Following DNA quality checks, samples are barcoded and ligated with sequencing adapters according to the kit instructions, with particular attention to minimizing DNA shearing and contamination. This workflow facilitates rapid, cost‐effective generation of multiplexed libraries suitable for real‐time sequencing and downstream genomic analyses.

### Materials

Extracted genomic DNA (gDNA) from Basic Protocols 2 and 3
Qubit dsDNA HS assay kit (Invitrogen, cat. no. Q32851)
AMPure XP beads (Beckman Coulter, cat. no. A63881)
NEBNext FFPE repair Mix (NEB)
NEBNext Ultra II end repair/dA‐tailing module (NEB, cat. no. E7546)
H_2_O, nuclease‐free (e.g., Thermo Fisher, cat. no. AM9937)
80% ethanol in nuclease‐free water, prepared fresh
NEB Blunt/TA ligase master mix (NEB, cat. no. M0367)
Native Barcoding Kit 24 V14 (Oxford Nanopore, cat. no. SQK‐NBD114.24)
NEBNext Quick Ligation module (NEB, cat. no. E6056)
50 mg/ml bovine serum albumin (BSA) (Invitrogen, cat. no. AM2616)

PPE including laboratory coat, nitrile gloves, protective eyewear
Qubit assay tubes (Invitrogen, cat. no. Q32856)
Pipettes: 0.1 to 2 µl, 0.5 to 10 µl, 1 to 20 µl, 10 to 100 µl, and 100 to 1000 µl; multichannel 0.5 to 10 µl and 1 to 20 µl
Filtered pipette tips corresponding to pipette sizes
Vortex mixer (e.g., VWR)
HulaMixer sample mixer (Thermo Fisher, cat. no. 15920D)
Qubit fluorometer (Thermo Fisher Scientific)
Ice bucket with ice
0.2‐ml thin‐walled PCR tubes (8‐strip preferred)
Microcentrifuge (e.g., Eppendorf 5424)
PCR thermal cycler (e.g., Bio‐Rad, T100)
1.5‐ and 2.0‐ml Eppendorf DNA LoBind tubes (e.g., VWR, cat. no. 76332‐068)
Magnetic rack (e.g., Agencourt magnetic stand, Beckman Coulter cat. no. A32782 or equivalent)
4°C refrigerator (for short‐term storage)
Computer with internet access
MinION instrument

#### Quantification of genomic DNA

1 Before starting, wear appropriate PPE and clean all laboratory surfaces to maintain a contamination‐free environment.
2 Prepare the Qubit working solution according to the manufacturer's instructions, using 190 µl for standard tubes and 198 µl for sample tubes. Add 10 µl of each Qubit standard (Standards 1 and 2) to their respective standard tubes.
3 For DNA samples, pipette 2 µl of each genomic DNA sample into individual sample tubes containing the working solution. Vortex each tube briefly for 3 s to mix thoroughly and incubate the tubes at room temperature (15° to 25°C) for 2 min to allow the dye to bind to DNA.
4 Measure DNA concentration using the Qubit fluorometer to ensure accurate quantification before proceeding with library preparation.

#### DNA repair and end‐preparation

5 Thaw AMPure XP beads (AXP) and DNA Control Sample (DCS) at room temperature. Mix by vortexing and keep on ice.
  For first‐time use of the DCS, dilute it by adding 105 µl of elution buffer (EB), mix gently, and centrifuge 5 s at 5000 × g, room temperature.
6 Prepare NEBNext FFPE DNA repair mix and Ultra II end prep module reagents on ice.
  Flick or invert to mix gently; do not vortex enzyme mixes. Before opening, centrifuge 5 s at 5000 × *g*, room temperature.
7 In a clean 0.2‐ml thin‐walled PCR tubes, prepare the DNA samples according to the number of barcodes.
  a. For >4 barcodes, use 400 ng of DNA per sample.
  b. For ≤4 barcodes, increase the input to 1000 ng per sample.
  c. Adjust the volume to 11 µl with nuclease‐free water, mix gently, and centrifuge 5 s at 5000 × *g*, room temperature.
8 A master mix containing all reagents (except the DNA samples) can be prepared to streamline the workflow for all samples included in the test (Table 1).
  To compensate for pipetting errors, prepare ∼10% more master mix than the required amount (e.g., if testing 10 samples, prepare enough master mix for 11 samples). Mix the master mix components thoroughly by gentle pipetting, followed by a brief centrifugation for 5 s at 5000 × g, room temperature, to collect the contents at the bottom of the tube.

**Table 1:** Recommended Volumes of Each Component for DNA Repair and End‐Preparation for Nanopore Library Preparation

Reagent	Volume per sample (µl)
Diluted DNA control sample (DCS)	1
NEBNext FFPE DNA repair buffer	0.875
Ultra II end‐prep reaction buffer	0.875
Ultra II end‐prep enzyme mix	0.75

9 Combine DNA samples with the master mix in the PCR tubes.
10 Incubate samples in a thermal cycler at 20°C for 5 min, then 65°C for 5 min.
11 Transfer each sample into clean 1.5‐ml DNA LoBind tube.

#### Bead cleanup

12 Resuspend AMPure XP beads by vortexing.
13 Add 15 µl beads to each end‐prep reaction in 1.5‐ml DNA LoBind tube from step 11; mix by flicking the tube.
14 Incubate for 5 min on HulaMixer at room temperature.
15 Centrifuge samples 5 s at 5000 × *g*, room temperature, pellet beads on a magnetic rack, and carefully remove the supernatant.
16 Prepare freshly made 80% ethanol in nuclease‐free water, allowing 400 µl per sample plus some excess.
17 Keep tubes on the magnet and wash beads twice with 200 µl of 80% ethanol.
  If the pellet was disturbed, wait for beads to pellet again before removing the ethanol.
18 Centrifuge 5 s at 5000 × *g*, room temperature, place on magnet, remove residual ethanol, and dry for ∼30 s (do not crack pellet).
  Allow it to dry for 30 s, but do not dry the pellets to the point of cracking.
19 Remove the tubes from magnetic rack and elute DNA in 10 µl nuclease‐free water. Centrifuge 5 s at 5000 × *g*, room temperature, and incubate for 2 min at room temperature.
20 Pellet beads until elute is clear and colorless, transfer eluate (10 µl) to clean 1.5‐ml tube. Discard the pelleted beads.
21 Quantify 1 µl of the eluate using Qubit. Store samples at 4°C if proceeding to barcoding later.
  Take forward an equimolar mass of each sample to be barcoded forward into the native barcode ligation step. However, you may store the samples at 4°C overnight.

#### Native barcode ligation and clean‐up

22 Thaw NEB Blunt/TA ligase master mix at room temperature, spin down, mix thoroughly by pipetting, and keep on ice.
23 Thaw the required Native Barcodes (NB01 to NB24), EDTA, and Short Fragment Buffer (SFB) at room temperature, mix, centrifuge 5 s at 5000 × *g*, room temperature, and store on ice.
  Only use one barcode per sample.
24 In clean 0.2‐ml PCR tube add the reagent in the order indicated in Table 2 per well.

**Table 2:** Recommended Volumes of Each Component for Native Barcode Ligation for Nanopore Library Preparation

Reagent	Volume (µl)
End‐prepped DNA	7.5
Native Barcode (NB01‐24)	2.5
Blunt/TA ligase master mix	10
Total	20

25 Mix gently, centrifuge 5 s at 5000 × *g*, room temperature, and incubate for 20 min at room temperature.
26 Add EDTA (2 µl for clear cap, 4 µl for blue cap) to each tube to stop reaction and mix thoroughly.
27 Pool all barcoded samples into a single 1.5‐ml DNA LoBind tube.
28 Resuspend the AMPure XP Beads by vortexing.
29 Add 0.4× volume of AMPure XP beads to the pooled reaction and mix gently.
30 Incubate for 10 min on a HulaMixer at room temperature.
31 Centrifuge 5 s at 5000 × *g*, room temperature, and pellet beads on the magnetic rack for 5 min. Remove supernatant carefully.
32 Keep the tube on the magnet and wash the beads twice with 700 µl SFB, taking care not to disturb the pellet. Remove and discard the buffer after each wash.
33 Centrifuge 5 s at 5000 × *g*, room temperature, place tube back on magnet, remove any residual buffer, and dry for ∼30 s.
34 Remove the tube from magnetic rack and resuspend pellet in 35 µl nuclease‐free water.
35 Incubate for 10 min at 37°C, flicking the tube every 2 min for 10 s to encourage DNA elution.
36 Pellet beads on a magnetic rack until the elute is clear and colorless.
37 Transfer 35 µl eluate to clean tube. Quantify using Qubit.
  Take forward the barcoded DNA library to the adapter ligation and clean‐up step. However, you may store the sample at 4°C overnight.

#### Adapter ligation and clean‐up

38 Thaw NEBNext Quick Ligation reaction module at room temperature (15° to 25°C) and centrifuge 5 s at 5000 × *g*, room temperature. Mix thoroughly using pipette (10 full‐volume pipette mix). Do not vortex T4 DNA ligase; keep on ice.
39 Thaw Native adapter, elution buffer, SFB, or Long Fragment Buffer (LFB) at room temperature and keep on ice.
  Use of SFB or LFB depending on fragment size. To enrich DNA fragments ≥3 kb, use LFB. To retain DNA fragments of all sizes, use SFB.
40 In a 1.5‐ml LoBind tube, combine the reagents inditcated inn Table 3 in order and mix by pipetting 10 times after each addition.

**Table 3:** Recommended Volumes of Each Component for Adapter Ligation for Nanopore Library Preparation

Reagent	Volume (µl)
Pooled barcoded sample	30
Native adapter (NA)	5
NEBNext quick ligation reaction buffer (5×)	10
Quick T4 DNA ligase	5
Total	50

41 Mix thoroughly and incubate for 20 min at room temperature.
42 Resuspend the AMPure XP beads by vortexing.
43 Add 20 µl AMPure XP beads to the ligation reaction and mix by pipetting.
44 Incubate for 10 min on a HulaMixer.
45 Spin down tube and pellet beads on magnetic rack. Keep the tube on the magnet and remove the supernatant.
46 Wash beads twice with 125 µl SFB to ensure the retention of all DNA molecules in the sample. Flick beads to resuspend, centrifuge 5 s at 5000 × *g*, room temperature, return tubes to magnet, and remove supernatant after each wash.
47 Centrifuge 5 s at 5000 × *g*, room temperature, place tubes back on the magnet, and remove residual supernatant.
48 Remove tube from magnetic rack and resuspend beads in 15 µl elution buffer.
49 Incubate for 10 min at 37°C, flicking every 2 min.
  Flick tube every 2 min for 10 s to encourage DNA elution.
50 Centrifuge 5 s at 5000 × *g*, room temperature, pellet beads on magnet until eluate is clear and colorless (at least 1 min), then transfer 15 µl eluate to a clean tube.
51 Quantify 1 µl of the library using Qubit fluorometer.
52 Adjust final library depending on DNA library fragment size. Prepare DNA in 12 µl EB (e.g., 100 fmol for <1 kb, 35 to 50 fmol for 1 to 10 kb, 300 ng for >10 kb). Store on ice or 4°C until loading.
  Based on the average fragment length and concentration obtained at this stage, you can calculate the amount of the final library to use in a 12‐µl reaction. For instance, if your final library concentration is 10 ng/µl and the average fragment length is 1.5 kb, you can use the NEB BioCalculator (see Internet Resources) to estimate molar concentration. Entering a DNA length of 1.5 kb and DNA mass of 10 ng gives ∼10.82 fmol. Therefore, using 4.5 µl of this library would provide ∼48.7 fmol. To achieve the required concentration in a total of 12 µl, mix 4.5 µl of library with 7.5 µl of elution buffer (EB).

#### Flow cell check, priming, and library loading (R10.4.1, FLO‐MIN114)

53 Go to EPI2ME downloads page (see Internet Resources) and select the MinKNOW installer based on your operating system then download and install the MinKNOW software (for first time user).
54 Ensure MinION Mk1B is connected to your computer via USB cable.
55 Log into MinKNOW software.
56 To perform a flow cell check, insert the flow cell firmly into the MinION Mk1B device. Apply gentle pressure to ensure proper seating. Place flow cell in MinION instrument towards the port showing 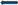 MinION.
57 Navigate to the Start page and click “Flow Cell Check” (top‐left) to view active pores. Click Start and the flow cell check will begin.
  MinKNOW will recognize the MinION Flow Cell type and IDs.
  For Flongle, fill in the flow cell ID manually.
58 Ensure ≥500 active pores before running the sequencing experiment. If you are using Flongle, make sure you have ≥500 active pores.
  If the flow cell has <500 active pores and is still within its expiry limit (3 months from the date you received it) or if a Flongle has <50 active pores and is within its expiry limit (1 month from the date you received it) contact ONT customer service (support@nanoporetech.com) to get a replacement.
  a. Thaw Sequencing Buffer (SB), Library Beads (LIB)/Library Solution (LIS), Flow Cell Tether (FCT), and Flow Cell Flush (FCF) at room temperature.
  b. Vortex, centrifuge 5 s at 5000 × *g*, room temperature, and keep on ice.
59 Prepare priming mix with BSA as indicated in Table 4.

**Table 4:** Recommended Volumes of Each Component for MinION Nanopore‐Flow Cell Priming

Reagent	Volume per flow cell (µl)
Flow cell flush (FCF)	1170
BSA at 50 mg/ml	5
Flow cell tether (FCT)	30
Total volume	1205

60 Slide priming port cover and draw back 20 to 30 µl to remove air bubbles.
  Set a P1000 pipette to 200 µl. Insert the tip into the priming port. Turn the wheel until the dial shows 220 to 230 µl, to draw back 20 to 30 µl, or until you can see a small volume of buffer entering the pipette tip. Take care when drawing back buffer from the flow cell. Do not remove more than 20 to 30 µl and always make sure that the array of pores is covered by buffer. Introducing air bubbles into the array can irreversibly damage pores.
61 Load 800 µl of the priming mix into the flow cell via the priming port, avoiding the introduction of air bubbles. Wait for 5 min.
62 Thoroughly mix the contents of the LIB by pipetting. It is vital that they are mixed immediately before use.
63 In a new 1.5‐ml DNA LoBind tube, prepare the loading mix as indicated in Table 5.

**Table 5:** Recommended Volumes of Each Component for MinION Nanopore‐Flow Cell Loading

Reagent	Volume per flow cell (µl)
Sequencing buffer (SB)	37.5
LIB mixed immediately before use	25.5
DNA library	12
Total	75

64 To complete the flow cell priming, gently lift the SpotON sample port cover to make the SpotON sample port accessible. Load 200 µl of the priming mix into the flow cell priming port (not the SpotON sample port), avoiding the introduction of air bubbles.
65 Mix the prepared library gently by pipetting up and down just prior to loading. Load 75 µl library into SpotON sample port dropwise.
66 Gently replace the SpotON sample port cover, making sure the bung enters the SpotON port and close the priming port and close priming port.
67 Place light shield on flow cell.
68 Close device lid. Set up sequencing run in MinKNOW.

#### Sequencing run set up

69 On MinKNOW home page, navigate to the Start page and click “Start Sequencing.”
70 In the positions tab, fill in the experiment name, sample ID, and select flow cell type and kit information (Flow Cell: FLO‐MIN114 and Kit: SQK‐NBD114.24).
71 Specify sequencing run length (e.g., 24 hr) and minimum read length or keep the default setting.
72 Click continue to analysis and choose basecaller model, select any barcoding options or keep the default setting. Click continue to output.
  For improved taxonomic resolution, it is recommended to set Basecalling to Super Accuracy, enable Trim Barcodes: ON, and set Barcode Both Ends: OFF. These settings enhance read quality and ensure more accurate identification during downstream analysis.
73 Specify your output data location, format or filtering options or keep default setting (e.g., /var/lib/minknow/data/). Click continue to final review.
74 Click “Start.”

## Basic Protocol 5 TAXONOMIC IDENTIFICATION USING EPI2ME DESKTOP APPLICATION

EPI2ME Desktop provides a user‐friendly platform for preliminary taxonomic identification of whole‐genome sequencing data. After downloading and installing the application for your operating system (Windows, macOS, or Linux), log in, install the wf‐metagenomics workflow, and import your FASTQ_pass reads. Run the workflow with default or customized parameters, and monitor progress until the HTML report is generated in the report tab.

### Necessary Resources

#### Hardware

Computer

##### Software

EPI2ME Desktop application

##### Files

FASTQ_pass reads (from Basic Protocol 4)

1 Download and install the EPI2ME Desktop application for your operating system (Windows, macOS, or Linux) from the EPI2ME downloads page (see Internet Resources).
2 Open the application and log in using your EPI2ME credentials.
3 Install the required workflow, such as wf‐metagenomics, for taxonomic identification.
4 Launch the workflow, choosing to run either locally or in the cloud.
5 Import your FASTQ_pass reads from the designated folder.
  You can upload entire folder instead of individual file.
6 Adjust workflow parameters if needed or run with default settings. Click “Run” and monitor the progress; the HTML report will be available in the report tab upon completion.

## TAXONOMIC IDENTIFICATION USING KRAKEN2 AND KRONA IN LINUX

This workflow enables comprehensive taxonomic analyses of nanopore sequencing data using Linux‐based pipelines, which is useful for custom databases (e.g., larger reference databases including plants to capture diverse microbiomes in the samples). Raw FASTQ files are processed through EPI2ME's metagenomics workflow, generating Kraken2 reports that can be visualized using Krona plots for species abundance per barcode. The protocol ensures reproducibility, traceability, and high‐resolution identification of plant pathogens.

### Necessary Resources

#### Hardware

Computer with ≥32 GB memory, ≥2 TB of SSD storage, USB Type‐C peripheral (USB 2.0 or higher), NVIDIA RTX 5090 Laptop GPU, and an Intel i7 CPU (≥12 cores)
*For more details, please refer to*: https://nanoporetech.com/ja/document/requirements/minion‐mk1d‐it‐reqs.

##### Software

Linux operating system

##### Files

FASTQ files (from Basic Protocol 4)

#### Data handling and transfer

1 Save/transfer base‐called FASTQ files from Linux computer to a Windows‐accessible folder if you are working in Windows subsystem for Linux.

#### Epi2me workflow setup (Linux terminal)

2 If running EPI2ME for the first time, create a dedicated environment.


conda create ‐n epi2me ‐c bioconda‐forge nextflow singularity

3 Activate the environment.


conda activate epi2me

4 Pull the EPI2ME metagenomics workflow.


nextflow pull epi2me‐labs/wf‐metagenomics

#### Run Epi2me metagenomics workflow

5 Execute the workflow.


nextflow run epi2me‐labs/wf‐metagenomics --fastq </path/to/barcodes/folder> ‐profile singularity

  Example:


nextflow run epi2me‐labs/wf‐metagenomics --fastq /mnt/plant_pathology/Students/Nanopore/fastq_pass_ Jan7_025/ ‐profile singularity

6 Workflow generates an output folder in the Linux home directory. Move this folder to a Windows‐accessible directory for downstream analysis (e.g., Epi2me_output_Jan7_025).
7 Open the folder corresponding to each barcode (e.g., barcode13) to view the number of reads assigned to each species.

#### Kraken2 and Krona visualization

8 Confirm the kraken2 database path (e.g., /qnap/databases/kraken2/k2_pluspfp_16gb_20241228.tar.gz).
9 Krona plots can be generated from Kraken2 output for each barcode. Run the following commands sequentially.


conda install ‐c bioconda krona
ktUpdateTaxonomy.sh
ktImportTaxonomy ‐t 5 ‐m 3 ‐o krona.html output/kraken2/*.kraken2.report.txt

  Example:


ktImportTaxonomy ‐t 5 ‐m 3 ‐o krona.html /mnt/plant_pathology/Students/Nanopore/Epi2me_output_Jan7_025/output/kraken2/*.kraken2.report.txt

10 Open the krona.html file from the Linux home directory or transfer it to your Windows subsystem of Linux for visualization if you are using Windows Subsystem Linux. Launch the plot in a browser and explore taxonomic levels interactively.

## Basic Protocol 6 BIOINFORMATICS ANALYSES FOR GENOME ASSEMBLY AND ANNOTATION

The whole‐genome obtained from pure bacterial cultures can be assembled and annotated to further understand their genome and for future comparative analyses.

### Necessary Resources

#### Hardware

Computer equipped with a Linux operating system

##### Software

dragonflye v1.2.1 (see Internet Resources)
bakta (see Internet Resources)

##### Files

FASTQ files generated by MinKnow software in ONT sequencing device

1 Concatenate all “pass” FASTQ files.


cd/mnt/plant_pathology/Students/Nanopore/fastq_pass_Sep24_024/barcode11
zcat *.fastq.gz | pigz ‐p 10 ‐ > concatenated_reads.fastq.gzip

  For concatenating files, you need to run this command in the directory with the passing reads for each barcode, e.g., to concatenate all the files for barcode 11. Repeat the process for each “pass” FASTQ files. Once “gzip” file is generated, you may need to change it to “gz” file.
2 Assemble bacterial genome using following commands in sequential order.


conda create ‐n dragonflye ‐c conda‐forge ‐c bioconda dragonflye
Conda activate dragonflye
dragonflye --cpus 20 --reads <path_to_concatenated_reads> --outdir <barcodeXX_assembly>

  Example:


dragonflye --cpus 20 --reads /mnt/plant_pathology/Students/Nanopore/concatenated.fastq.files.Sep24_024/barcode13/concatenated_reads.fastq.gz --outdir barcode13_assembly

  Run commands in new terminal or in base.
3 Output will be generated now in HOME directory (of linux) with the name “BARCODE13” i.e., same name of input file.
  Open file “dragonflye.log” for assembly results.
4 Bacterial genome annotation using bakta.


conda create ‐n bakta ‐c conda‐forge ‐c bioconda bakta
conda activate bakta
bakta_db download --output /qnap/databases/bakta --type full

  Example:


bakta --db /qnap/databases/bakta/db --output barcode13_assembly/annotation --threads 20 /mnt/plant_pathology/Students/Nanopore/Assembly_results_Jan07_025/barcode13_assembly/contigs.fa

  Open annotation‐stats.tsv file to view annotation result.

## REAGENTS AND SOLUTIONS

### King's B (KB) medium

*This medium is a semi‐selective medium to isolate pseudomonads from plant samples*.

To prepare KB medium for culturing *Pseudomonas* spp., mix 20 g of proteose peptone (Thermo Fisher, cat. no. 211693), 10 ml glycerol (Thermo Fisher, cat. no. 15514011), 1.5 g of dipotassium phosphate (K_2_HPO_4_) (Sigma‐Aldrich, cat. no. P3786), 1.5 g of magnesium sulfate heptahydrate (MgSO_4_·7H_2_O) (Sigma‐Aldrich, cat. no. 230391), and 15 g agar (if preparing solid medium; Thermo Fisher, cat. no. A10752.36) in 1 L distilled water (King et al., 1954). Add the components to a screw‐cap bottle containing a magnetic stir bar and gently mix on a magnetic stirrer until fully dissolved. The initial pH of the solution is typically ∼7.5; adjust this to pH 7.2 using 1 M HCl. Sterilize the medium by autoclaving at 121°C for 15 to 20 min. After autoclaving, allow the medium to cool to ∼50°C before pouring into sterile Petri dishes under aseptic conditions or in a BSC. For optimal growth and visualization of fluorescent *Pseudomonas* colonies under UV light, it is recommended to use freshly prepared solidified KB agar plates.

## COMMENTARY

### Background Information

Traditional plant pathogen diagnostics rely heavily on culture‐based phenotyping systems (e.g., BIOLOG) and single‐gene PCR assays (Gerin et al., 2019; Lee et al., 2023; Weisburg et al., 1991). While these approaches remain valuable, they often lack the taxonomic resolution required to distinguish closely related members of the pseudomonads. Universal 16S rRNA primers, although widely used, are not capable of reliably differentiating pathogenic PSSC members from closely related non‐pathogenic strains such as *Pseudomonas fluorescens*. Moreover, these methods are time‐consuming, labor‐intensive, challenges of isolating pathogenic strains of *Pseudomonas* spp. during hot and dry season and require interpretation by trained specialists.

Recent advancements in portable sequencing technologies, particularly the MinION nanopore sequencer, have significantly transformed the landscape of plant disease diagnostics. Although MinION was initially adopted for plant virus detection (Della Bartola et al., 2020; Martins et al., 2021; Vazquez‐Iglesias et al., 2022), it has since been applied to the detection of fungal pathogens (Hassan et al., 2022; Yang et al., 2022) and bacterial species (Faino et al., 2021). The platform offers numerous advantages, including real‐time sequencing with immediate data analysis, the ability to generate long reads ideal for genome assembly, and high sequencing yields ≥10 Gbp well above the size of most bacterial genomes (Haveman & Schuerger, 2022; Shivashakarappa et al., 2022). Additionally, MinION's portability and simplified library preparation protocols make it especially suited for on‐site and remote applications. With its raw read accuracy reaching >99% with new chemistry and latest basecalling models, this technology emerging as a promising tool for real‐time and high‐resolution pathogen detection.

The development of a MinION‐based diagnostic tool capable of sequencing directly from infected tissue introduces a valuable opportunity for simultaneous detection of multiple known and unknown pathogens in a single assay. Currently, no universal molecular protocol exists for the direct identification of *P. syringae* species group members infecting blueberries. This study demonstrates that by integrating MinION nanopore sequencing with user‐friendly analysis pipelines such as EPI2ME, accurate and real‐time identification of bacterial canker pathogens in blueberries can be achieved. The approach is applicable to both cultured isolates and DNA extracted directly from infected plant tissue, providing a robust framework for implementing nanopore sequencing in routine diagnostics and surveillance programs. The workflow is compatible with the portable Oxford Nanopore MinION platform and can be further optimized for field‐deployable applications. Simplified library preparation steps, minimal equipment requirements, and real‐time data acquisition provide opportunities for adaptation to on‐site pathogen detection, and with additional optimization, this protocol may support fully mobile diagnostic use cases in field or resource‐limited settings. Furthermore, this methodology can be extended to other high‐value horticultural crops, enhancing early detection and mitigation strategies for emerging or quarantine pathogens.

### Critical Parameters

#### Sample collection and selection considerations

Consistency in sample collection is crucial to minimizing technical variability in Nanopore‐enabled MinION sequencing diagnostics. In field conditions, the timing of sample collection can influence pathogen detectability, especially during warmer months when certain pathogens (e.g., *Pseudomonas*) may be less readily isolated using culture‐based techniques. However, as shown in this study, the ability to identify pathogens directly from host tissue using nanopore‐enabled MinION sequencing provides a valuable advantage.

Samples should be stored immediately at 4°C, ideally using portable coolers during transport, to preserve DNA integrity and reduce degradation. Tissue selection is also critical: sampling from the leading edge of symptomatic areas—where tissue is half‐healthy and half‐diseased, particularly in regions showing wet or oozing bacterial symptoms—can significantly improve both isolation and molecular detection outcomes.

#### DNA extraction from culture and kit selection

The choice of DNA extraction method directly impacts DNA yield, downstream sequencing quality, and taxonomic identification. In this study, the InstaGene Matrix kit was selected for extracting DNA from cultures due to its rapid protocol, low cost, and consistently reliable sequencing performance. While manual extraction methods remain practical for small‐scale studies, the adoption of automated systems can minimize batch effects and improve throughput. Laboratories handling large numbers of samples should consider automated extraction to save time and ensure greater consistency across runs.

#### Inclusion of controls

Controls are essential at each step of the workflow to ensure data validity and trace contamination. Positive controls, e.g., known pathogens, serve to verify the accuracy of DNA extraction, amplification, and sequencing. Negative controls, including blank extractions and non‐template library preparations, are necessary to identify potential contamination sources. For accredited diagnostic settings, such as those laboratories operating under International Organization for Standardization or any standards council or any laboratory accreditation and assessment services standards, it is imperative that all sequencing assays are validated using these controls to establish detection thresholds and monitor for external contamination.

#### Custom reference databases in bioinformatics analysis

Accurate bioinformatic analysis requires careful consideration of reference database composition. In metagenomic studies, especially when using nanopore sequencing, a broad range of microbial taxa may be detected. Limiting the analysis to only a subset of bacterial species can introduce significant bias. Instead, custom reference databases that include a wide range of bacterial taxa, as well as host plant sequences, can provide a more comprehensive and unbiased picture of the microbial community and support accurate pathogen identification.

#### Interpretation and reporting of results

Interpretation of nanopore‐generated data presents one of the greatest challenges in diagnostic applications. Nanopore sequencing platforms like MinION often detect multiple microbes, increasing the risk of false positives or misclassification (Khan et al., 2018). This is particularly true when closely related species share conserved genetic regions or when data quality is low or when sample was not processed with caution and aerosol contamination may happen.

Distinguishing between pathogenic and non‐pathogenic taxa requires expert interpretation and may necessitate additional confirmatory tests, such as fulfilling Koch's postulates. Misclassification can be especially problematic when dealing with low‐abundance pathogens or asymptomatic hosts (Wu et al., 2015). Therefore, diagnostic reports must include appropriate disclaimers and clearly define criteria for further action in cases of ambiguous identification.

Fortunately, user‐friendly platforms, such as EPI2ME and Krona plots, which were utilized in this study, offer streamlined analysis pipelines. These tools improve accessibility, reduce analytical errors, and support the accurate identification of pathogens in complex metagenomic datasets.

### Troubleshooting

Nanopore sequencing workflows for plant‐associated bacterial diagnostics are prone to several technical issues that can compromise data quality or lead to failed runs. In Table 6, we summarize common problems encountered during DNA extraction, library preparation, and sequencing, along with their likely causes and practical solutions.

**Table 6:** Troubleshooting Guide for Nanopore Sequencing in Plant Diagnostics

Problem	Possible cause	Solution
Low DNA yield (Qubit) <20 ng/µl	Incomplete or impure extraction	Use an alternative extraction kit; include a purification step (e.g., column‐based or magnetic bead cleanup)
Low library recovery	Incorrect AMPure XP beads ratio	Ensure beads are thoroughly resuspended; use ≥0.4:1 bead‐to‐sample ratio
Low recovery after end prep	Ethanol concentration <70% during wash	Use freshly prepared 70% ethanol for wash steps
Low pore count at run start	Air bubbles in the flow cell	Carefully remove air bubbles before priming/loading
Pore count discrepancy after loading	Improper flow cell insertion	Re‐insert the flow cell; confirm correct temperature and seating
Pore count discrepancy after loading	Library contaminants (e.g., polysaccharides)	Re‐extract or purify DNA using a clean‐up kit (e.g., PowerClean Pro)
Script failed (MinKNOW)	Software crash	Restart MinKNOW and the host computer; collect and review error logs if issue persists
Inactive or unavailable pores	Air bubbles during priming	Follow ONT best‐practice videos; use slow, consistent pipetting
	Plant polysaccharide carryover	Use a plant‐specific DNA extraction kit or perform additional clean‐up
	Other contaminants	Re‐extract DNA using optimized protocols for plant material

Low DNA yield often results from incomplete lysis or the presence of contaminants that inhibit nucleic acid recovery. Impurities, e.g., polysaccharides and phenolic compounds frequent in plant tissues, can also impact pore activity, or compromise downstream reactions. Issues in library recovery are commonly associated with incorrect AMPure XP bead handling, particularly improper bead‐to‐sample ratios, or use of suboptimal ethanol concentrations during wash steps.

Discrepancies in pore counts, either at the beginning of a run or between flow cell checks, may stem from air bubbles introduced during flow cell priming, incorrect insertion of the flow cell, or contaminants that block the pores. Additionally, software crashes in MinKNOW, the control software for ONT devices, may occur sporadically and are often resolved through system restarts and log reviews.

By recognizing and addressing these common challenges proactively, users can significantly improve the reliability and reproducibility of nanopore sequencing for plant pathogen diagnostic applications.

### Understanding Results

When isolating members of plant pathogenic pseudomonads from infected plant tissues (Basic Protocol 1), cultures should display characteristic colony morphology on selective medium (King's B medium). Selecting a single, well‐isolated colony for downstream processing minimizes the risk of mixed cultures and ensures the purity required for high‐quality sequencing. DNA extracted from either pure isolates (Basic Protocol 2) or infected plant tissue (Basic Protocol 3) should be of sufficient purity to support nanopore library preparation. Kits such as the Bio‐Rad InstaGene Matrix or the Qiagen DNeasy Plant Mini Kit typically yield DNA compatible with long‐read sequencing; however, quantification by Qubit fluorometry is preferred, as spectrophotometric methods can overestimate yield in low‐biomass or plant‐rich samples. Because plant tissues often contain polysaccharides, phenolics, and other inhibitors, low or inconsistent DNA yields may occur. In such cases, implementing column‐ or bead‐based cleanup steps or switching extraction chemistries can improve purity and performance. Although Oxford Nanopore recommends ∼400 ng DNA input, libraries generated from as little as ∼180 ng have produced successful sequencing output when protocols are optimized to reduce DNA loss.

During library preparation (Basic Protocol 4), users should expect measurable DNA at each quantification checkpoint (post‐DNA repair, post‐barcoding, and after adapter ligation), which confirms that long‐fragment integrity has been preserved throughout the workflow. The Native Barcoding Kit 24 V14 (SQK‐NBD114.24) enables multiplexing of up to 24 samples, and clean barcode separation should be observed when cross‐contamination is limited, and DNA is handled gently to avoid mechanical shearing. Reduced read lengths, insufficient barcode separation, or anomalously low library yields may indicate over‐fragmentation or improper cleanup steps.

Sequenced samples are expected to produce sequencing data of sufficient quality for downstream analyses. Average read lengths of 1.5 to 2.0 kb are anticipated, with a substantial proportion of reads exceeding this range after quality control (2.0 to 2.7 kb). Basecalling was performed using MinKNOW with the super‐accurate model enabled, and the majority of reads across sequencing runs consistently achieve Q scores ≥20, with >90% of reads meeting this threshold. Compared with commonly used nanopore library preparation methods, this protocol emphasizes gentle handling, avoidance of mechanical shearing, and bead‐based cleanup conditions optimized to retain longer DNA fragments. These steps are intended to preserve DNA integrity and support improved read length distributions.

Taxonomic identification using the EPI2ME Desktop application (Basic Protocol 5) should result in clear species‐level classifications from FASTQ_pass reads processed with the wf‐metagenomics workflow. The interactive HTML reports allow visualization of relative abundances for each barcode and should consistently identify Psc when isolates or tissue samples contain this pathogen. Compared to direct tissue sequencing, sequencing from pure bacterial culture generally exhibits higher relative abundance values for the species of Psc, reflecting the reduced background signal from host DNA and competing microbiota.

For users applying Linux‐based workflows (Support Protocol), Kraken2 paired with Krona visualization should provide hierarchical, high‐resolution taxonomic assignments. The resulting interactive plots allow drilling down from the phylum to the species level and are particularly informative for distinguishing Psc from co‐occurring bacterial taxa in mixed infections. Consistency between EPI2ME and Kraken2/Krona outputs, particularly the repeated identification of Psc at high relative abundance, serves as an internal check on sequencing quality and taxonomic accuracy.

Genome assembly and annotation (Basic Protocol 6) should yield assemblies with metrics reflecting typical Psc genome structure. High‐quality assemblies are expected to include values for total genome size, contig number, longest contig, mean contig length, and N50 consistent with long‐read or hybrid assembly performance. For Psc isolates, total genome size should fall within an anticipated range of 5.8 to 6.4 Mb, accompanied by annotation results listing predicted protein‐coding sequences (CDS), pseudogenes, tRNA, rRNA, and ncRNA genes, as well as GC content. These assembly statistics, when in the expected ranges, can be used to confirm sample identity, assess completeness, and support later comparative or pangenomic analyses.

### Time Considerations

The duration of the nanopore sequencing pipeline depends on whether the process begins with pure bacterial cultures or directly from infected plant tissues. When starting with pure cultures, the initial step of bacterial isolation from symptomatic tissue takes ∼2 days. During this time, symptomatic samples are plated, incubated, and assessed for colony morphology, with careful selection of a single colony to ensure purity and accuracy in downstream analyses.

Following isolation, genomic DNA can be extracted using different kits. For pure cultures, the InstaGene Matrix protocol requires ∼40 min. When DNA is extracted directly from infected plant tissue using the DNeasy Plant Mini Kit, the process takes ∼1 hr. After extraction, DNA is quantified using Qubit fluorometry, which typically takes an additional 15 min.

Library preparation, which includes DNA repair, barcoding, and adapter ligation, requires between 3 to 5 hr depending on the number of samples processed. The Native Barcoding Kit 24 V14 supports up to 24 samples per run and includes optional overnight pauses, which provide flexibility in handling multiple or complex sample sets. Once libraries are prepared, flow cell priming and loading is relatively quick and takes 10 to 15 min, assuming the absence of air bubbles or other complications.

Sequencing on the Oxford Nanopore MinION platform proceeds in real time and can be run for 24 to 48 hr, depending on the desired read depth and resolution. Throughout the run, basecalling occurs automatically, with data stored in the FASTQ_pass format for downstream analysis.

Taxonomic identification using the EPI2ME Desktop application is rapid and user‐friendly. The default wf‐metagenomics workflow generates results within ∼10 min. When using larger or custom reference databases, the runtime may extend to 15 to 30 min depending on computational resources. Visualization of the results using Krona plots, which allow interactive taxonomic exploration down to the species level, takes ∼5 min per sample and supports high‐resolution assessment of complex microbial communities. Genome assembly and annotation can be completed in few hours to days depending on computing resources.

In total, the entire pipeline—from culture isolation, DNA extraction, quantification, library preparation, sequencing, taxonomic identification, and genome assembly and annotation—can be completed within 4 to 5 days. For workflows that bypass culturing and extract DNA directly from infected tissue, the total time can be reduced to 2 to 3 days. The comparison of diagnostic methods and estimated total time required is shown in Table 7. This represents a significant improvement over traditional diagnostics, offering near real‐time pathogen identification and enabling more timely and informed disease management decisions in agricultural settings.

**Table 7:** Comparison of Diagnostic Methods and Estimated Time Required for These Methods

Diagnostic method	Key steps involved	Estimated time required	Notes
Traditional culture‐based diagnostics	Isolation from symptomatic tissue, culturing, phenotypic and/or molecular identification	1‐2 weeks	Time‐consuming due to slow growth and multiple confirmatory steps
Nanopore sequencing (pure bacterial cultures)	Culture isolation, DNA extraction, library preparation, sequencing, taxonomic identification, genome assembly	4‐5 days	Includes ∼2 days for bacterial isolation; provides species‐level identification and genomic information
Nanopore sequencing (directly from infected plant tissue)	Direct DNA extraction, library preparation, sequencing, taxonomic identification, genome assembly	2‐3 days	Culturing step is bypassed, significantly reducing turnaround time
EPI2ME taxonomic identification	wf‐metagenomics workflow and result visualization (Krona plots)	10‐30 min	Rapid, user‐friendly workflow; runtime may vary with database size and computing resources

### Cost Considerations

The approximate per‐sample cost of MinION sequencing in this study was $200 to $215 CAD, compared with ∼$35 CAD per isolate for BIOLOG‐based identification and ∼$105 CAD per isolate for PCR with Sanger sequencing (Table 8). Reagents pricing was assessed on August 07, 2024, and subject to change.

**Table 8:** Cost Comparison Between MinION Sequencing and Conventional Methods

Method	Procedures	Per‐sample cost (CAD)	Comments/Challenges
BIOLOG	Culture, microplate, misc.	$35	Species‐level ID not always possible; multiple plate readings required
PCR + Sanger	Culture, DNA extraction, PCR, sequencing	$105	Multiple PCRs may be required; includes controls
MinION (culture‐based)	Culture, DNA extraction, library prep	$215	Flow cell reuse limited in QA labs; research settings may allow up to 24 samples/run
MinION (direct tissue)	DNA extraction, library prep	$205	Avoids culture step; enables direct detection from infected tissue

Although MinION sequencing is relatively expensive, its superior resolution and ability to detect pathogens without culturing or unculturable pathogens. In addition, identification of bacteria (e.g., BIOLOG‐based) is not widely accepted. Actual costs are influenced by factors, such as sample throughput, protocol optimization, reagent sourcing, and sequencing depth. Per‐sample costs can be reduced substantially through multiplexing, e.g., using 24 to 96 barcodes per run. While sequencing costs have decreased in recent years, routine implementation of whole‐genome or metagenomic sequencing in diagnostic laboratories may not be cost‐effective unless high‐throughput sample processing is feasible. However, the cost of MinION‐based detection could be justifiable for the detection of rapidly evolving, multiple pathogens associated with disease complex or unculturable pathogens. Early and accurate pathogen detection enables timely intervention, supporting effective disease management and the deployment of best‐practice mitigation strategies.

### Author Contributions

**Rishi Burlakoti**: Conceptualization; funding acquisition; project administration; resources; supervision; writing—original draft; writing—review and editing. **Sanjib Sapkota**: Conceptualization; data curation; formal analysis; methodology; writing—original draft; writing—review and editing. **Pragyan Burlakoti**: Conceptualization; supervision; writing—review and editing. **Mark Lubberts**: Formal analysis; software; writing—review and editing. **Amy Novinscak**: Methodology; writing—review and editing. **Harvinder Bennypaul**: Conceptualization; writing—review and editing.

### Conflict of Interest

The authors declare that the research was conducted in the absence of any commercial or financial relationships that could be construed as a potential conflict of interest.

## Data Availability

The data that support the findings of this study are available from the corresponding author upon reasonable request.
